# pH Dependence of Amyloid‐β Fibril Assembly Kinetics: Unravelling the Microscopic Molecular Processes

**DOI:** 10.1002/anie.202210675

**Published:** 2022-10-27

**Authors:** Yao Tian, John H. Viles

**Affiliations:** ^1^ Department of Biochemistry School of Biological and Behavioural Sciences Queen Mary University of London London UK

**Keywords:** Alzheimer's Disease, Amyloid, Isoelectric Point, Kinetics, Molecular Mechanism

## Abstract

Central to Alzheimer's disease (AD) is the assembly of the amyloid‐beta peptide (Aβ) into fibrils. A reduction in pH accompanying inflammation or subcellular compartments, may accelerate fibril formation as the pH approaches Aβ’s isoelectric point (pI). Using global fitting of fibril formation kinetics over a range of pHs, we identify the impact net charge has on individual fibril assembly microscopic rate constants. We show that the primary nucleation has a strong pH dependence. The titration behaviour exhibits a mid‐point or p*K*_a_ of 7.0, close to the p*K*_a_ of Aβ histidine imidazoles. Surprisingly, both the secondary nucleation and elongation rate constants are pH independent. This indicates the charge of Aβ, in particular histidine protonation, has little impact on this stage of Aβ assembly. These fundamental processes are key to understanding the forces that drive the assembly of Aβ into toxic oligomers and fibrils.

## Graphical abstract

Globally fitting different kinetic models of fibril formation, over a range of pHs, reveals the key microscopic molecular processes of amyloid assembly. Primary nucleation is driven by a loss of net charge, while surprisingly, both secondary nucleation and elongation rates are independent of pH.

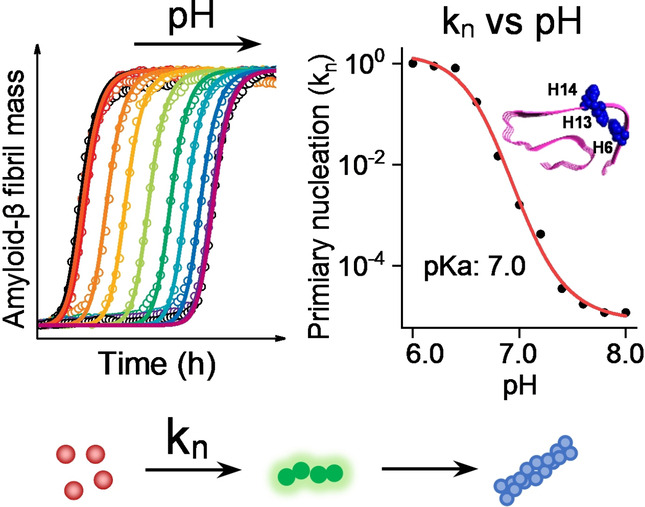

Alzheimer's disease (AD) is the most common dementia currently responsible for 46 million cases worldwide.^[1]^ Substantial genetic evidence^[2]^ supports the amyloid cascade hypothesis which states a key early event in AD pathology is the self‐association and accumulation of Aβ peptide in to oligomers and fibrillary assemblies, observed in senile plaques of AD patients.^[3]^ This Aβ peptide, is typically 40 or 42 amino acids in length and causes cellular membrane disruption^[4]^ and a loss of cellular homeostasis which may lead to cell death and dementia.^[3]^

AD is linked with inflammation which can cause acidic micro‐environments,^[5]^ and Aβs assembly into fibrils is sensitive to pH. Reduction from physiological pH to a pH closer to Aβs isoelectric point (pI) of 5.3 will reduce its solubility, and so increase self‐association and the rate of amyloid formation.^[6]^ Aβ accumulates in endo‐lysosomal vesicles where the rate of oligomer formation is accelerated by the low pH.^[6c]^ What is not understood is how pH can affect the individual microrate constants associated with various molecular processes of amyloid assembly.

A nucleated polymerization reaction describes the process of Aβ monomer assembly into amyloid fibrils.^[7]^ In vitro, kinetic traces of the reaction have a sigmoidal appearance, with an initial slow lag‐phase in which many nucleating oligomeric Aβ seeds will form.^[8]^ Often the fibril specific fluorescent dye thioflavin‐T (ThT) is used to monitor macroscopic amyloid fibril formation.^[9]^

A method to extract specific microrate constants from the macroscopic kinetic behaviour has been developed by globally fitting the kinetic traces over a range of Aβ concentrations.^[10]^ In particular, microrate constants for: the primary nucleation (*k_n_*); secondary fibril surface catalysed nucleation (*k*_2_); and the elongation rate on the ends of growing fibrils can be obtained (*k*_+_).[^10^, ^11^] The surface of the fibrils can act as a template for secondary nucleation,[^11b^, ^12^] which is distinct from the exposed ends of an elongating fibril.^[13]^ Here we show that only primary nucleation has a strong pH dependence, while fibril surface catalysed secondary nucleation and elongation are independent of pH.

Solubilization of Aβ at pH 10, followed by size exclusion chromatography leads to essentially monomeric Aβ, Figure S1. After surveying several buffering conditions, we found 50 mM phosphate buffer and 50 mM NaCl produced a consistent set of kinetic curves over a range of pH values (pH 6.0–8.0), for both Aβ40 and Aβ42 (5 μM). Fibril growth kinetic curves for Aβ40 and Aβ42 between pH 6 to 8 are shown in Figure 1A–B; S2 and S3. Four traces are shown for each pH value, monitored by fibril specific ThT florescence. A single representative trace is shown in Figure 1C and D for eleven pH values, between pH 6 and 8. The sigmodal fibril formation kinetic curves have been fitted^[14]^ to determine the *t*_1/2_ (the time to reach half maximal ThT fibril signal), together with *t*_lag_ (the time to reach the end of the lag‐phase) and fibril growth time, *t*_growth_ (the time for the signal to go from 10 % to 90 % ThT maximum signal, the elongation phase). pH‐dependent behaviour is shown for *t*_1/2_, Figure 1E and similarly for *t*_lag_ Figure S4, this data fits well to a Henderson–Hasselbalch pH dependant titration curve. The mid‐point of this pH dependant transition (p*K*_a_) is 7.0 for both Aβ40 and Aβ42, supplemental Table S1. In contrast, the *t*_growth_ values (slope of the kinetic trace) are completely independent of pH, between pH 6 and 8, Figure 1F. To a large extent the ThT fluorescence signal has been shown to be proportional to the total amount of fibril mass.^[9]^ A plot of ThT maximal signal versus pH indicates a constant fluorescence signal, independent of pH, see Figure S5.

**Figure 1:**
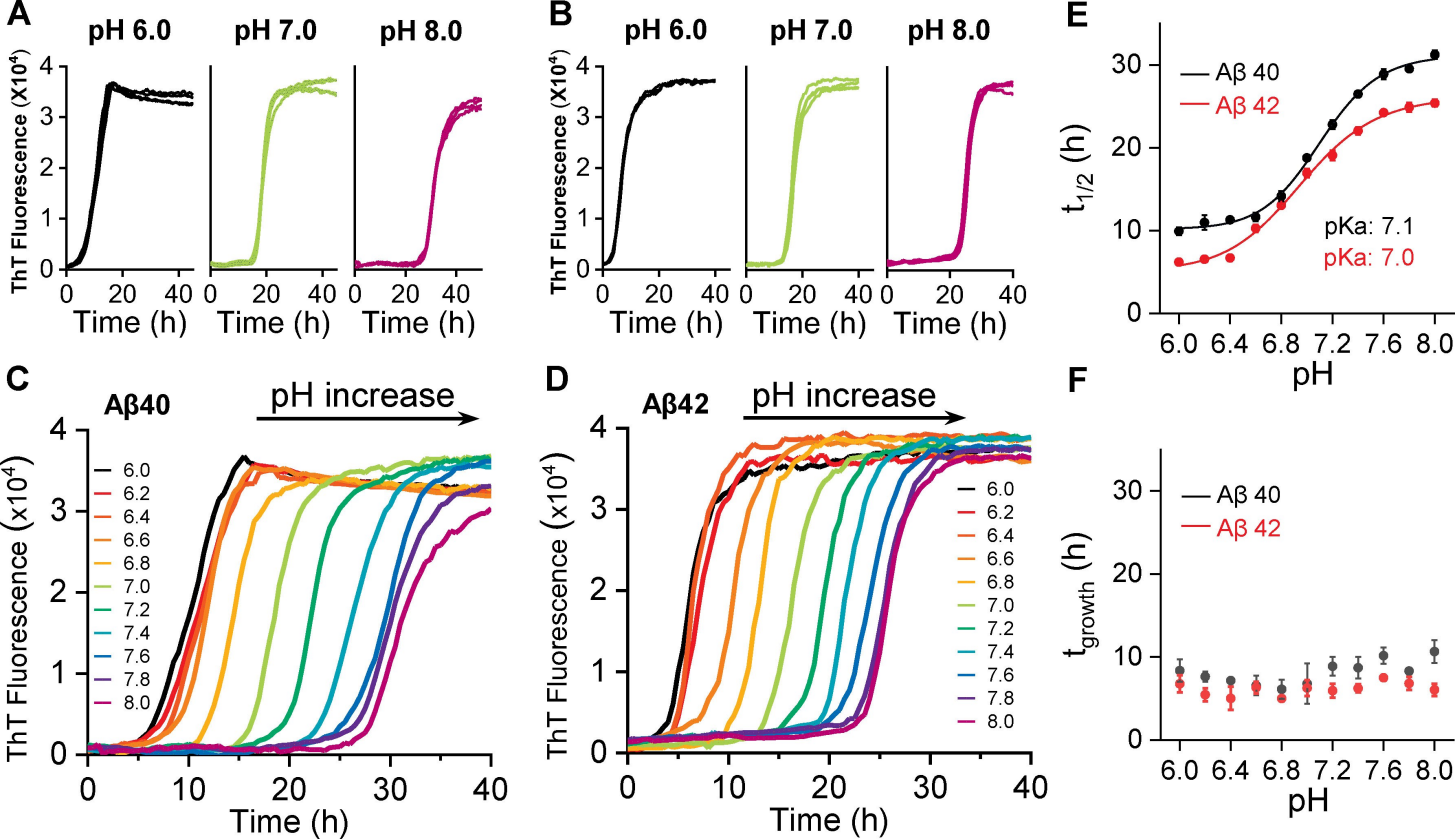
pH‐dependent fibril formation kinetics of Aβ40 and Aβ42. ThT kinetic traces (*n*=4) at pH 6.0, 7.0 and 8.0 for Aβ40 (A) and Aβ42 (B), see also supplemental S2 and S3. Single representative traces for Aβ40 (C) and Aβ42 (D) between pH 6.0–8.0, from left (black, pH 6.0) to right (purple, pH 8.0). E) Plots of *t*_1/2_ versus pH, with p*K*_a_ fitted. F) Plots of growth‐time versus pH; error bars are standard error of the mean (SEM) from four replicates.

To investigate which of the microscopic kinetic assembly processes are most dependant on pH we used “AmyloFit” a global kinetic fitting program for the analysis of amyloid formation kinetics.^[10]^ The data set of kinetic curves are obtained with constant Aβ concentration but over a large range of pHs. A similar approach has been used to investigate how inhibitors of fibril formation effect individual microrate constants.^[15]^ When our kinetic traces were globally fitted to this kinetic model, primary nucleation rate constants (*k_n_*) were varied, while secondary rate constants (*k*_2_) and elongation rate constants (*k*_+_) remained fixed. The experimental data‐set could be closely fitted to the simulated kinetic curves, Figure 2A. In contrast, if the primary nucleation rate constant (*k_n_*) was unaltered and secondary (*k*_2_) or elongation (*k*_+_) rate constants were permitted to vary, the global fit to the experimental data was poor, Figure 2B and C. This data suggests that changes in pH between 6 and 8 have a profound impact on molecular processes associated with primary nucleation (Figure 2D), while secondary nucleation and elongation rates are largely unchanged over this pH range. The data fitted in Figure 2 is for Aβ42, identical behaviour is observed for Aβ40, as shown in Figure S6.

**Figure 2:**
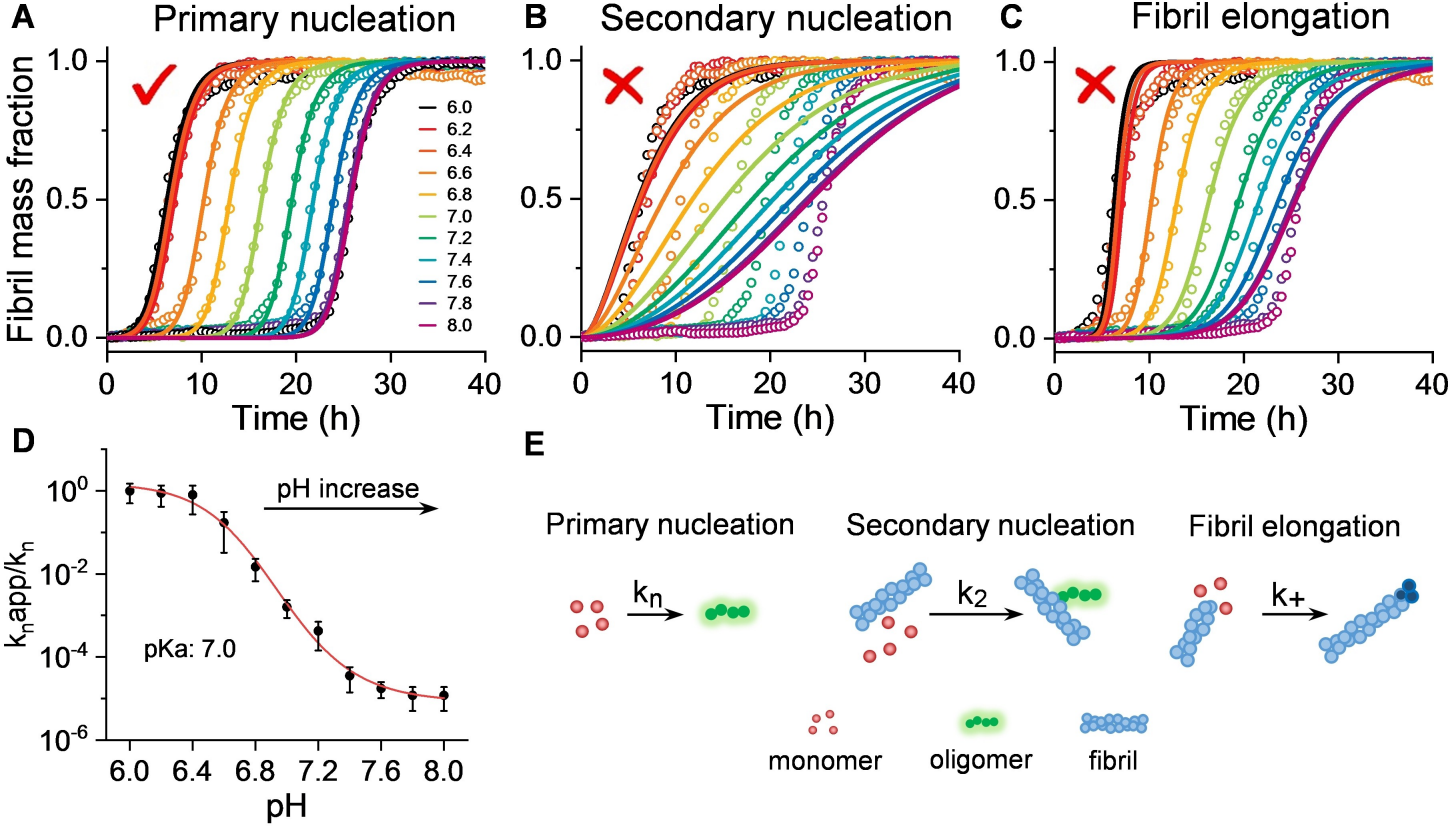
pH effects primary nucleation processes of Aβ42 aggregation. A–C) Kinetics profiles of Aβ42 (5 μM) at pH 6.0–8.0, from left (black, pH 6.0) to right (purple, pH 8.0). The solid lines represent global fits of the kinetic traces when only primary nucleation (A), secondary nucleation (B) and fibril elongation (C) rate constants are altered to globally fit pH dependent traces. (D) Change in primary nucleation rate constants (*k_n_*) versus pH, derived from global fits in Figure 2A, error bars are SEM from four replicates. E) Schemes of the microscopic steps for primary nucleation, secondary nucleation, and fibril elongation.

To test the above assertion, we obtained a set of seeded fibril growth measurements over the same pH range by adding a 10 % (monomer equivalent) fibril seed, generated at the different pH values. Adding fibril seeds have the effect of circumventing primary nucleation, in these seeded experiments the kinetic traces are dominated by the effect of elongation and particularly secondary nucleation from the surface of the fibril seeds.^[11b]^ Remarkably, in these seeded experiments the pH dependence of the fibril growth rate is completely lost, Figure 3 and S7. There is no change in the *t*_1/2_, *t*_lag_ or *t*_growth_ values between pH 6 and 8. This data strongly supports the analysis in Figure 2, indicating that primary nucleation is very sensitive to pH, while the opposite is the case for secondary fibril surface catalysed nucleation and elongation which must dominate in these seeded experiments.

**Figure 3:**
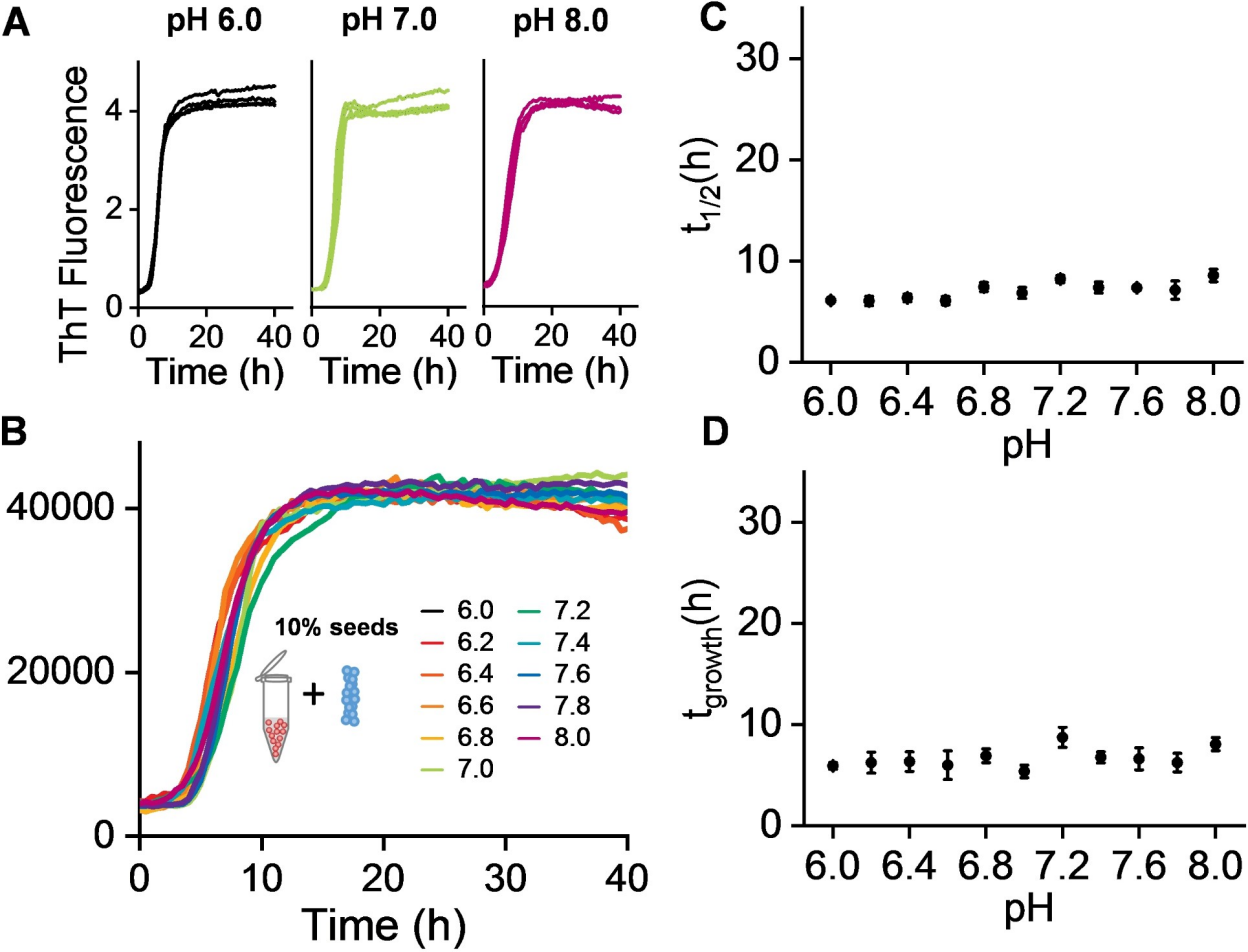
Seeded fibril formation is pH independent. This seeded kinetics indicates secondary nucleation (*k*_2_) and elongation (*k*_+_) rates are independent of pH. A) ThT kinetic traces (*n*=4) at pH 6.0, 7.0 and 8.0 for Aβ40 (5 μM) with 10 % fibril seed, see also Figure S7. B) Single representative traces for Aβ40 between pH 6.0–8.0. C) *t*_1/2_ versus pH, error bars are SEM from 4 replicates. D) Growth‐time versus pH.

Finally, we were interested in how the pH might affect the morphology of amyloid fibrils, and in particular, the extent of periodic twists in the fibrils. Charge on the protofibril surface might affect the packing of these to form fibrils and so alter the twist morphology. The node‐to‐node period in the twists is very consistent for Aβ40; 141+/−15 nm, Figure 4A, C and S8. A much tighter twist is observed for Aβ42 with a periodicity of 31+/−5 nm, Figure 4B, D and S9. Comparison of the fibril twists period over a range of pH values, 6.0 to 8.0 indicates the morphology of fibrils is indistinguishable over this range, for both Aβ40 and Aβ42, with little variation in the periodicity of the twist between pH 6 and 8. The diameter of fibrils was also measured and found to be similar over all pHs for both Aβ40 ca. 14 nm and Aβ42 ca. 11 nm.

**Figure 4:**
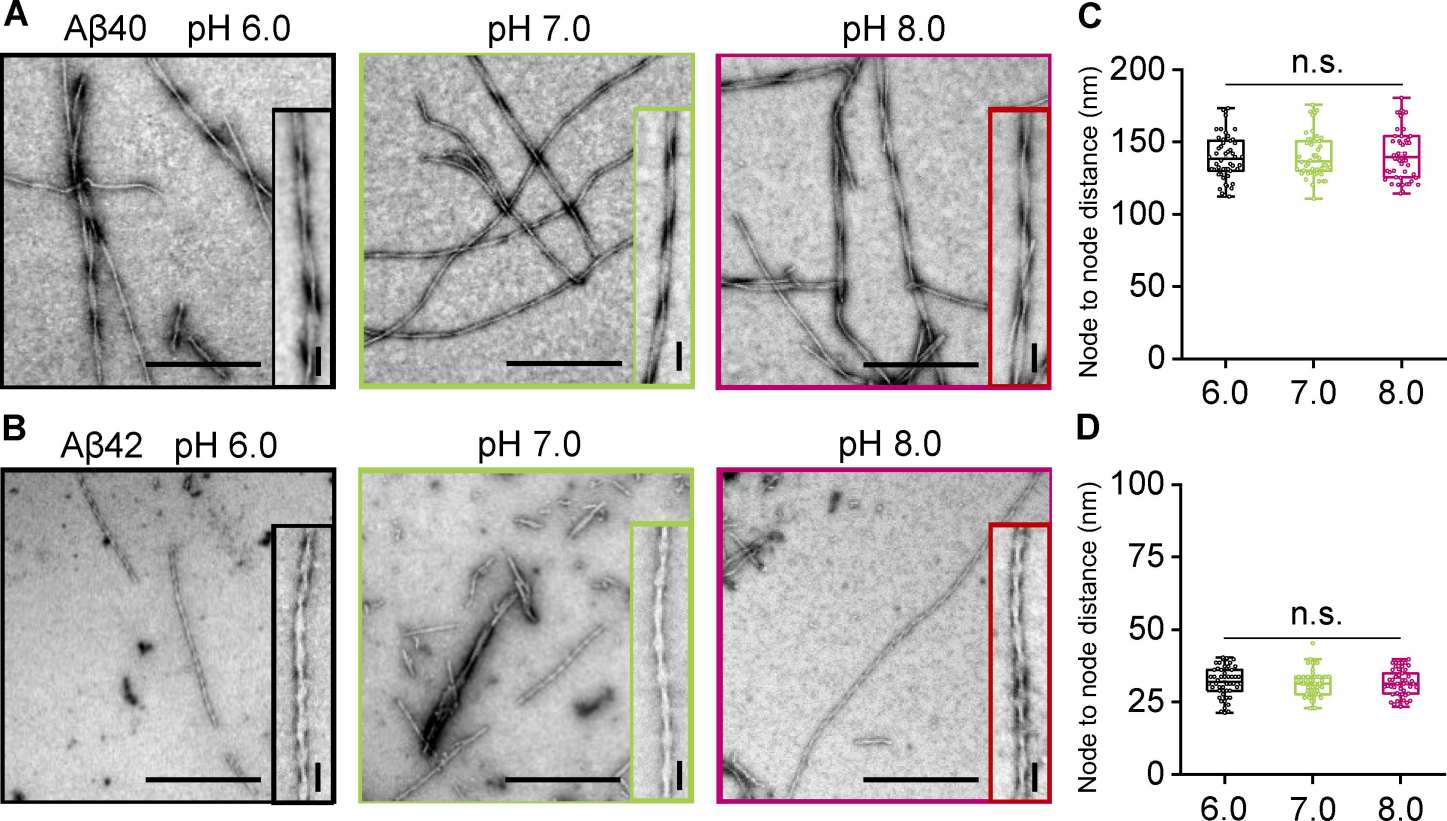
pH has negligible effect on the morphology of Aβ40 and Aβ42 fibrils. Negatively stained TEM fibril images produced at pH 6.0, 7.0 and 8.0 for Aβ40 and Aβ42 (A, B). Further examples are shown Figure S8 and S9. Scale bars: 500 nm; inset 50 nm. Node‐to‐node distance of Aβ40 (C) and Aβ42 (D) fibril twists at pH 6.0, 7.0 and 8.0. N=50 individual fibrils are measured per condition. The median value is shown as a line, boxes are 25–75 % of median values, error bars are 1.5 interquartile range.

Self‐association of Aβ monomer is perhaps the first step in amyloid assembly, and will be driven by a whole range of molecular interactions; these include the hydrophobic effect and electrostatic attraction/repulsion. Aβ40 and Aβ42 have a pI of 5.3, at higher pHs Aβ is negatively charged and this raises its overall solubility.^[6]^ As the pH is lowered from 8 to 6, Aβ’s histidines protonate (His6, His13 and His14), consequently Aβ becomes more neutrally charged. The p*K*_a_ of all three histidine side chains in monomeric Aβ has been determined to be 6.7,^[16]^ which is typical for a histidine in an unstructured peptide. The importance of the titrating imidazole in Aβ fibril formation is emphasized by the pH dependence of the *t*_1/2_ and *t*_lag_ and *k_n_* for both Aβ40 and Aβ42, whose midpoint of this transition (p*K*_a_) is 7.0 which is close to the three histidine p*K*_a_′s, Table S1. The N‐terminal amino group will also titrate (p*K*_a_ 7.9) which explains the slight shift in p*K*_a_ from 6.7 to 7.0. Indeed, the mean p*K*_a_ of the three histidine's plus the N‐terminus is equal to 7.0.

During the process of primary nucleation at least two Aβ molecules need to associate, our data indicates this will more readily occur if the net charge of Aβ is close to zero, so there is little electrostatic repulsion, and so intermolecular self‐association can be driven by hydrophobic contacts. Perhaps surprisingly, once the primary nucleation occurs, the rate of elongation on the ends of fibrils is independent of the net charge of Aβ, particularly the charge of the histidine imidazole rings. Similarly, surface catalysed secondary nucleation is independent of the histidine protonation state. This suggests the site of nucleation on the surface of fibrils is unaffected by protonation of the histidine's. Despite the sensitivity of sequence to cross‐seeding,^[17]^ point mutations of hydrophobic residues on the fibril surface have limited impact on secondary nucleation, which highlights the generality of the surface catalysed effect.^[12]^

The change in protonation state of the histidine sidechains does not appear to affect the fibril morphology, Figure 4. Other alterations in the charge on the side‐chain caused by point mutations found in familial AD, do influence fibril structure and the fibril twist periodicity.[^17^, ^18^] However, most structures of Aβ indicate the histidine's are not in the structured core of the fibril,^[19]^ as highlighted in supplemental Figure S10.

pH Dependent behaviour of other amyloid forming proteins have been reported,^[20]^ although individual microscopic rate constants have not been obtained. Much of this data suggests the protein's pI is an important determinant of fibril kinetics.^[21]^

In conclusion, globally fitting microrate constants for fibril assembly suggests the net charge and loss of electrostatic repulsion of Aβ has a major impact of self‐association during the formation of primary nuclei but has negligible influence on fibril elongation and fibril surface catalysed nucleation. In vivo, acidic micro‐environments such as those found at the surface of anionic phospholipid membranes, sub‐cellular compartments such as the endosome and lysosome,^[6c]^ or those induced by inflammation,^[5]^ might trigger the initial primary nucleation leading to the amyloid cascade. The design of inhibitors of amyloid assembly that add negative charge to Aβ might be an effective inhibitor of primary nucleation.

## Conflict of interest

The authors declare no conflict of interest.

## Supporting information

As a service to our authors and readers, this journal provides supporting information supplied by the authors. Such materials are peer reviewed and may be re‐organized for online delivery, but are not copy‐edited or typeset. Technical support issues arising from supporting information (other than missing files) should be addressed to the authors.

Supporting Information

## Data Availability

The data that support the findings of this study are available from the corresponding author upon reasonable request.
